# Supplementary material for: Post-transcriptional knowledge in pathway analysis increases the accuracy of phenotypes classification
Source: Oncotarget. 2016 Jun 2;7(34):54572–82. doi: 10.18632/oncotarget.9788 (PMC5342365; doi:10.18632/oncotarget.9788)
Supplement: Supplementary file 1 [file oncotarget-07-54572-s001.pdf]

Post-transcriptional knowledge in pathway analysis increases the accuracy of phenotypes classification

SUPPLEMENTARY DATA

INTRODUCTION

Here we provide the results of our comprehensive experimental analysis of MITHrIL. First we report the complete performance evaluation (Figure 1-3) of *MITHrIL* applied to tumors extracted from TCGA, by comparing it to *SPIA* [1], *PARADIGM* [2], *and Micrographite* [3]. Each curve in Figures 1-3 shows receiver operating characteristic (ROC) curves which are indicative of the ability of the algorithm to distinguish real pathways from decoy ones, using the ranking computed by each method. For each curve in figures 1-3 we also provide their respective average area (AUC) values in Supplementary Table S1.

Supplementary Tables S2-8 report all the results of the classifications performed with the algorithm *PAMr* [4]. The results are provided in the form of confusion matrices.

Finally, in figure 4 we show an example of pathway endpoint selection. All pathway endpoins are marked in red. Next to each endpoint, the KEGG database reports its direct or inderect effect on the cellular phenotype.

REFERENCES

1. Tarca AL, Draghici S, Khatri P, Hassan SS, Mittal P, Kim J-S, Kim CJ, Kusanovic JP, Romero R. A novel signaling pathway impact analysis. *Bioinformatics*. 2009;25:75-82.
2. Vaske CJ, Benz SC, Sanborn JZ, Earl D, Szeto C, Zhu J, Haussler D, Stuart JM. Inference of patient-specific pathway activities from multi-dimensional cancer genomics data using PARADIGM. *Bioinformatics*. 2010;26:i237-i245. doi:10.1093/bioinformatics/btq182.
3. Calura E, Martini P, Sales G, Beltrame L, Chiorino G, D'Incalci M, Marchini S, Romualdi C. Wiring miRNAs to pathways: a topological approach to integrate miRNA and mRNA expression profiles. *Nucl Acids Res*. 2014;42:e96-e96. doi:10.1093/nar/gku354.
4. Tibshirani R, Hastie T, Narasimhan B, Chu G. Diagnosis of multiple cancer types by shrunken centroids of gene expression. *Proceedings of the National Academy of Sciences*. 2002;99:6567-6572.

SUPPLEMENTARY FIGURES AND TABLES

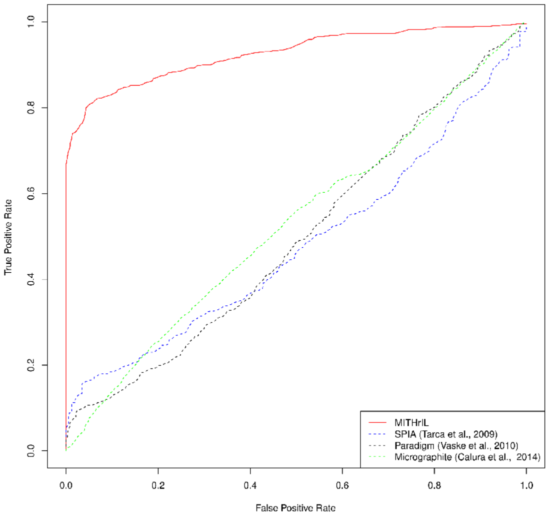

BLCA

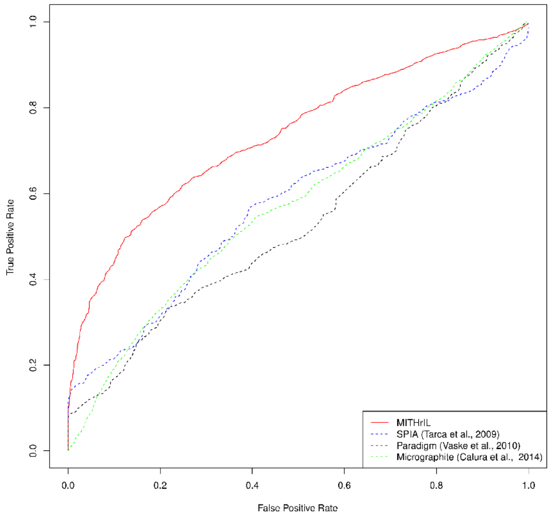

BRCA

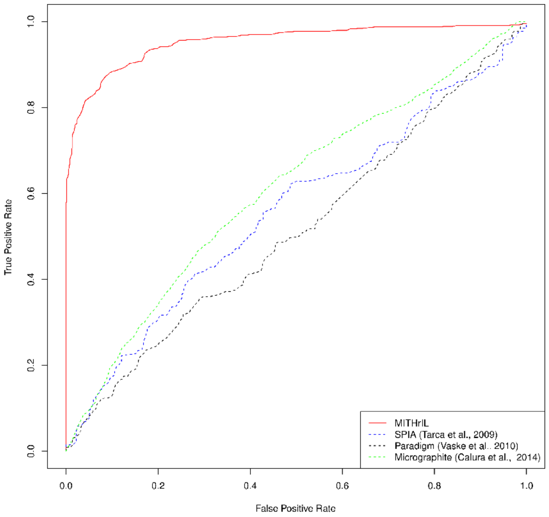

COAD

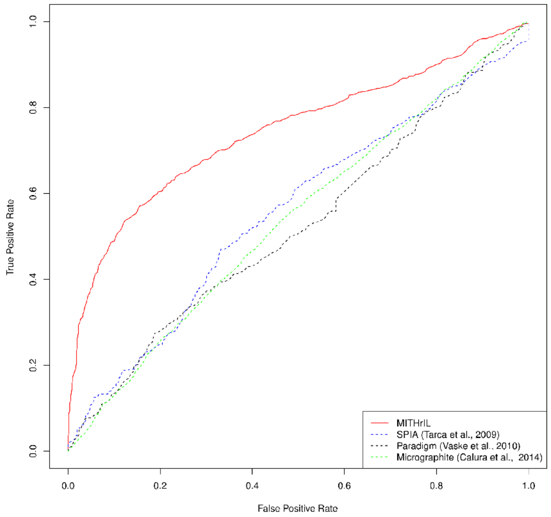

KICH

Supplementary Figure S1: Comparison between MITHrIL , SPIA [1], paradigm [2], and micrographite [3] by means of decoy pathways on four cancer datasets (BLCA, BRCA, COAD, KICH). Decoy pathways were created by assigning random nodes to each pathway. MITHrIL , SPIA and Paradigm were used to compute the perturbation of every pathway. Each line shows the receiver operating characteristic (ROC) curves for distinguishing real pathways from decoys, using the pathway ranking.

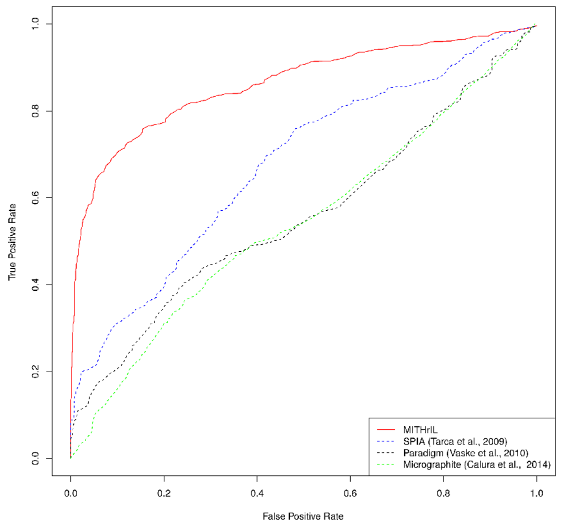

KIRC

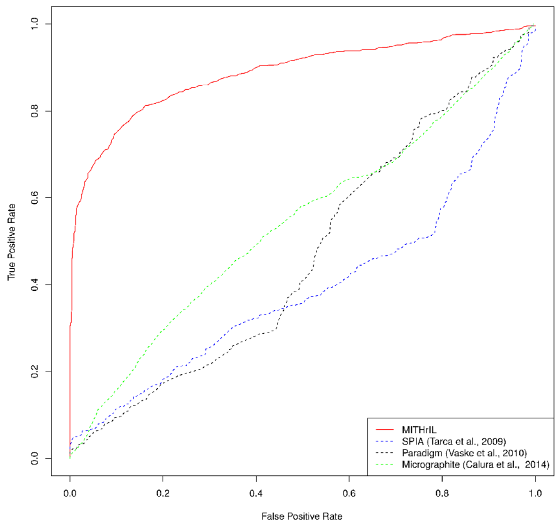

LUAD

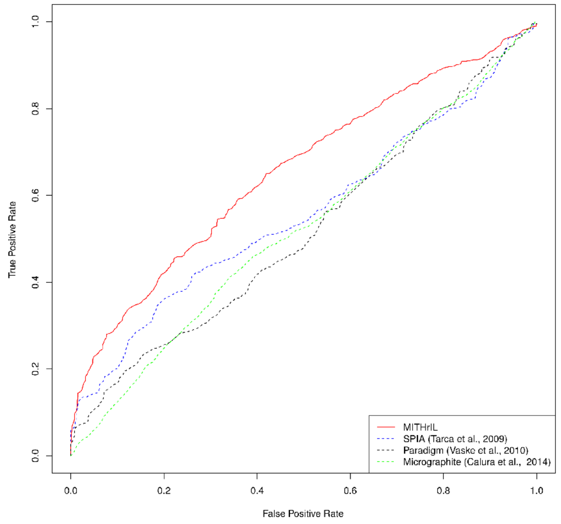

LUSC

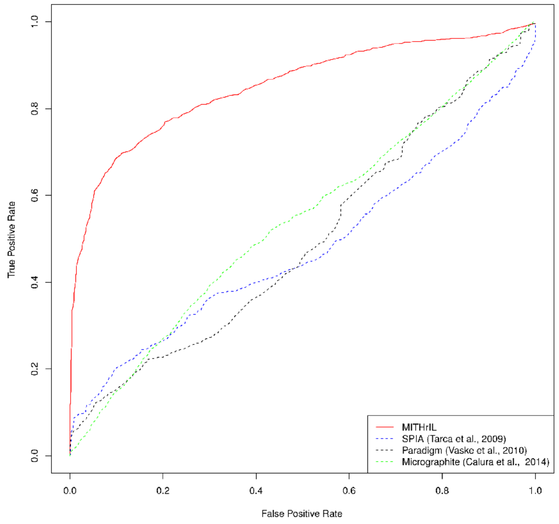

PRAD

Supplementary Figure S2: Comparison between MITHrIL , SPIA [1], paradigm [2], and micrographite [3] by means of decoy pathways on four cancer datasets (KIRC, LUAD, LUSC, PRAD). Decoy pathways were created by assigning random nodes to each pathway. MITHrIL , SPIA and Paradigm were used to compute the perturbation of every pathway. Each line shows the receiver operating characteristic (ROC) curves for distinguishing real pathways from decoys, using the pathway ranking.

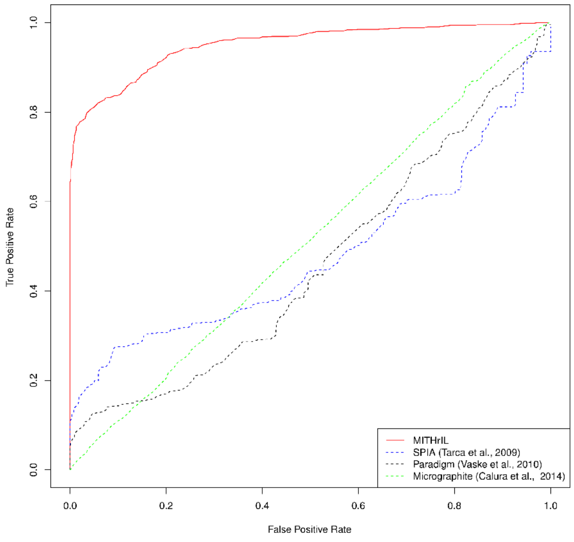

READ

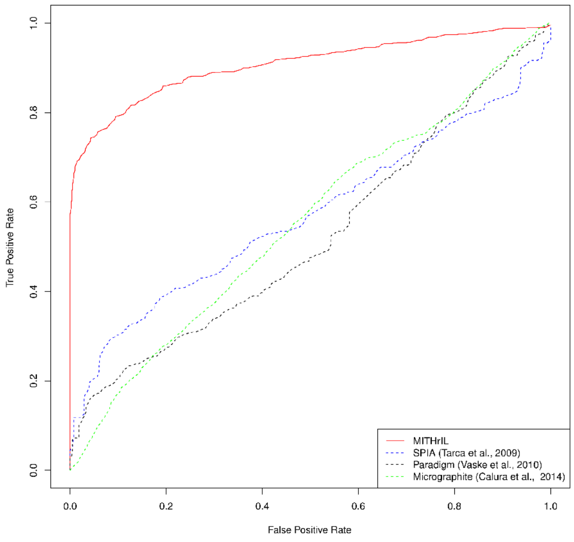

UCEC

Supplementary Figure S3: Comparison between MITHrIL , SPIA [1], paradigm [2], and micrographite [3] by means of decoy pathways on two cancer datasets (READ, UCEC). Decoy pathways were created by assigning random nodes to each pathway. MITHrIL , SPIA and Paradigm were used to compute the perturbation of every pathway. Each line shows the receiver operating characteristic (ROC) curves for distinguishing real pathways from decoys, using the pathway ranking.

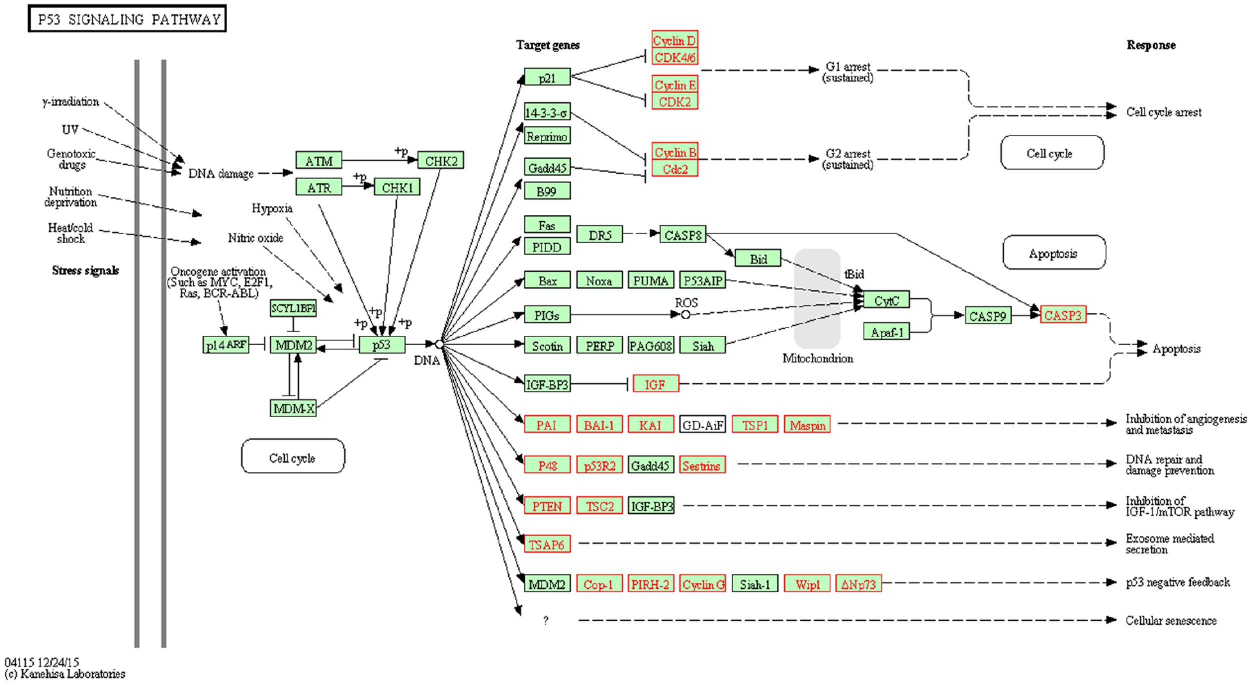

Supplementary Figure S4: Example of pathway after applying the endpoint selection algorithm based on a DFS visit of the pathway graph. Each endpoint is marked in red. Next to each endpoint the KEGG database shows the direct or indirect effect on the cellular phenotype.

Supplementary Table S1: Average areas under the curve (AUC) of figure 2 computed for all cancer datasets. best results are in bold

	Data set										
**Algorithm**	**BLCA**	**BRCA**	**COAD**	**KICH**	**KIRC**	**LUAD**	**LUSC**	**PRAD**	**READ**	**UCEC**	**Overall**
**MITHrIL**	**0.9263**	**0.7362**	**0.9535**	**0.7448**	**0.8628**	**0.8875**	**0.6553**	**0.8521**	**0.9447**	**0.9074**	**0.84706**
**SPIA**	0.4813	0.5801	0.5585	0.5572	0.6708	0.3967	0.5527	0.4806	0.4809	0.5709	0.53297
**PARADIGM**	0.4973	0.5335	0.5121	0.5227	0.5587	0.4693	0.5180	0.4987	0.4594	0.5224	0.50921
**Micrographite**	0.5246	0.5678	0.6049	0.5374	0.5402	0.5396	0.5187	0.5373	0.5108	0.5500	0.54313

Supplementary Table S2: Confusion matrix for the classification obtained by applying PAMR [4] algorithm to the Log-Fold-Change of differentially expressed genes for the 10 types of cancer in our dataset

	BLCA	BRCA	COAD	KICH	KIRC	LUAD	LUSC	PRAD	READ	UCEC	Class error rate
**BLCA**	**187**	2	0	0	0	0	0	3	0	1	3.11%
**BRCA**	0	**630**	0	0	0	0	0	12	0	0	1.86%
**COAD**	0	0	**380**	0	0	0	0	1	8	0	2.31%
**KICH**	0	0	0	**64**	2	0	0	0	0	0	3.03%
**KIRC**	2	0	0	5	**217**	0	0	2	0	0	3.12%
**LUAD**	0	0	0	0	0	**369**	16	3	0	0	4.89%
**LUSC**	0	0	0	0	0	15	**232**	0	0	0	6.07%
**PRAD**	0	0	0	0	0	0	0	**191**	0	0	0.00%
**READ**	0	0	5	0	0	0	0	0	**145**	0	3.33%
**UCEC**	4	0	0	0	0	0	0	0	0	**227**	1.73%
**Overall error rate**											2.90%

Supplementary Table S3: Confusion matrix for the classification obtained by applying PAMR [4] algorithm to MITHrIL perturbation values computed for 3165 pathway endpoints using our expression data set

	BLCA	BRCA	COAD	KICH	KIRC	LUAD	LUSC	PRAD	READ	UCEC	Class error rate
**BLCA**	**190**	0	0	0	0	0	0	3	0	0	1.55%
**BRCA**	0	**635**	0	0	0	0	0	7	0	0	1.09%
**COAD**	0	0	**389**	0	0	0	0	0	0	0	0.00%
**KICH**	0	0	0	**66**	0	0	0	0	0	0	0.00%
**KIRC**	0	0	0	2	**219**	0	0	3	0	0	1.79%
**LUAD**	0	0	0	0	0	**381**	0	7	0	0	1.80%
**LUSC**	0	0	0	0	0	3	**244**	0	0	0	1.21%
**PRAD**	0	0	0	0	0	0	0	**191**	0	0	0.00%
**READ**	0	0	0	0	0	0	0	0	**150**	0	0.00%
**UCEC**	0	0	0	0	0	0	0	0	0	**231**	0.00%
**Overall error rate**											0.90%

Supplementary Table S4: Confusion matrix for the classification obtained by applying the PAMr [4] algorithm to MITHrIL perturbation values computed for 3165 pathway endpoints using our expression data set

	BLCA	BRCA	COAD	KICH	KIRC	LUAD	LUSC	PRAD	READ	UCEC	Class error rate
**BLCA**	**188**	0	0	0	0	0	0	3	0	2	2.59%
**BRCA**	0	**629**	0	0	0	0	0	13	0	0	2.02%
**COAD**	0	0	**389**	0	0	0	0	0	0	0	0.00%
**KICH**	0	0	0	**66**	0	0	0	0	0	0	0.00%
**KIRC**	0	0	0	3	**218**	0	0	3	0	0	2.68%
**LUAD**	0	0	0	0	0	**380**	0	8	0	0	2.06%
**LUSC**	0	0	0	0	0	5	**242**	0	0	0	2.02%
**PRAD**	0	0	0	0	0	0	0	**191**	0	0	0.00%
**READ**	0	0	0	0	0	0	0	0	**150**	0	0.00%
**UCEC**	0	0	0	0	0	0	0	1	0	**230**	0.43%
**Overall error rate**											1.40%

MITHrIL was run without the integration of miRNA annotation in order to assess their contribution to the classification model.

Supplementary Table S5: Confusion matrix for the classification obtained by applying PAMR [4] algorithm to MITHrIL Accumulator values computed for all pathways using our expression data set

	BLCA	BRCA	COAD	KICH	KIRC	LUAD	LUSC	PRAD	READ	UCEC	Class error rate
**BLCA**	**168**	2	0	1	0	8	0	14	0	0	12.95%
**BRCA**	6	**558**	0	33	0	35	0	6	0	4	13.08%
**COAD**	0	0	**386**	2	0	1	0	0	0	0	0.77%
**KICH**	0	2	0	**63**	0	0	0	1	0	0	4.54%
**KIRC**	1	0	0	6	**211**	1	0	5	0	0	5.80%
**LUAD**	2	2	0	0	0	**371**	0	13	0	0	4.38%
**LUSC**	2	7	0	3	0	0	**234**	1	0	0	5.26%
**PRAD**	2	0	0	3	0	0	0	**186**	0	0	2.61%
**READ**	0	0	1	0	0	0	0	0	**149**	0	0.66%
**UCEC**	1	0	0	0	0	7	0	2	0	**221**	4.32%
**Overall error rate**											6.40%

Supplementary Table S6: Confusion matrix for the classification obtained by applying PAMR [4] algorithm to SPIA [1] Accumulation values computed for all pathways using our expression data set

	BLCA	BRCA	COAD	KICH	KIRC	LUAD	LUSC	PRAD	READ	UCEC	Class error rate
**BLCA**	**97**	0	0	0	0	0	0	14	9	73	49.74%
**BRCA**	1	**589**	4	0	0	0	0	34	14	0	8.25%
**COAD**	0	0	**389**	0	0	0	0	0	0	0	0.00%
**KICH**	0	0	1	**64**	0	0	0	1	0	0	3.03%
**KIRC**	0	0	0	5	**218**	0	0	0	1	0	2.67%
**LUAD**	0	0	0	0	0	**377**	0	8	3	0	2.83%
**LUSC**	2	0	0	0	0	7	**237**	1	0	0	4.04%
**PRAD**	3	0	6	1	0	2	0	**132**	46	1	30.89%
**READ**	0	0	0	0	0	0	0	0	**150**	0	0.00%
**UCEC**	0	0	0	0	0	0	0	3	0	**228**	1.29%
**Overall error rate**											8.80%

Supplementary Table S7: Confusion matrix for the classification obtained by applying PAMR [1] algorithm to PARADIGM [2] scores computed for all pathways using our expression data set

	BLCA	BRCA	COAD	KICH	KIRC	LUAD	LUSC	PRAD	READ	UCEC	Class error rate
**BLCA**	**34**	44	2	5	0	36	2	6	0	64	82.38%
**BRCA**	22	**173**	29	67	14	46	4	76	0	211	73.05%
**COAD**	7	19	**261**	6	2	3	14	24	0	53	32.90%
**KICH**	0	12	2	**45**	5	0	0	1	0	1	31.81%
**KIRC**	4	13	0	12	**145**	0	4	12	0	34	35.26%
**LUAD**	13	20	27	13	1	**138**	24	72	0	80	64.43%
**LUSC**	1	8	22	1	0	59	**70**	6	0	79	71.54%
**PRAD**	0	4	3	18	2	2	0	**154**	0	7	18.94%
**READ**	2	10	91	2	1	3	6	7	**5**	23	96.66%
**UCEC**	4	86	1	2	0	14	0	0	0	**124**	46.32%
**Overall error rate**											57.60%

Supplementary Table S8: Confusion matrix for the classification obtained by applying the PAMr [1] algorithm to PARADIGM [2] scores computed for 3165 pathway endpoints using our expression data set

	BLCA	BRCA	COAD	KICH	KIRC	LUAD	LUSC	PRAD	READ	UCEC	Class error rate
**BLCA**	**189**	1	0	0	2	0	0	0	0	1	2.08%
**BRCA**	2	**627**	0	0	0	0	2	10	0	1	2.34%
**COAD**	0	0	**377**	0	1	0	0	0	11	0	3.10%
**KICH**	0	0	0	**64**	2	0	0	0	0	0	3.03%
**KIRC**	0	0	0	5	**217**	0	0	2	0	0	3.13%
**LUAD**	0	0	0	0	0	**367**	18	3	0	0	5.41%
**LUSC**	0	0	0	0	0	17	**229**	0	0	0	6.91%
**PRAD**	0	0	0	0	1	0	0	**189**	0	0	0.52%
**READ**	0	0	6	0	0	0	0	0	**144**	0	4.00%
**UCEC**	1	0	0	0	0	0	1	0	0	**229**	0.09%
**Overall error rate**											3.20%
